# Extraction of high-quality RNA from mouse pancreatic tumors

**DOI:** 10.1016/j.mex.2020.101163

**Published:** 2020-11-26

**Authors:** Kranthi Kumar Chougoni, Steven R. Grossman

**Affiliations:** a C. Kenneth and Diane Wright Center for Clinical and Translational Research, Virginia Commonwealth University, Richmond, VA 23298, United States; b Department of Internal Medicine, Virginia Commonwealth University, Richmond, VA 23298, United States; c VCU Massey Cancer Center, Virginia Commonwealth University, Richmond, VA 23298, United States

**Keywords:** Pancreatic cancer, RNA extraction, High quality RNA, Ribonuclease, Pancreas, RIN

## Abstract

Extraction of high-quality RNA from pancreatic tumors for sequencing purposes is technically challenging, as the pancreas is an organ rich in ribonucleases. The majority of the established RNA isolation protocols for use with primary pancreatic tissue involve perfusion of RNA stabilizing reagent into the pancreatic tissue to protect RNA integrity before extraction. However, the additional time needed for this procedure can actually lead to further RNA degradation. We optimized a protocol suitable for high quality RNA isolation from mouse pancreatic tumors that is a simple, fast, and inexpensive modification of existing methods, combining the use of liquid nitrogen and guanidinium thiocyanate-chloroform extraction. Through this procedure, the mean RNA Integrity Number value obtained for RNA isolated from pancreatic tumors was 9.0, and was reproducibly suitable for RNAseq and qPCR.

• a protocol suitable for high quality RNA isolation from mouse pancreatic tumors as well as normal pancreas
• combining the use of liquid nitrogen and guanidinium thiocyanate-chloroform extraction

## Graphical abstract

**Figure:**
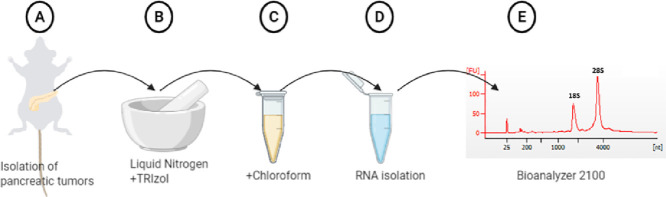

Specifications table

**Table:** 

Subject Area:	Biochemistry, Genetics and Molecular Biology
More specific subject area:	Cancer Biology
Method name:	Extraction of high-quality RNA from mouse pancreatic tumors
Name and reference of original method:	Li, D., Ren, W., Wang, X. et al. A Modified Method using TRIzol^Ⓡ^ Reagent and Liquid Nitrogen Produces High-Quality RNA from Rat Pancreas. Appl Biochem Biotechnol **158,** 253–261 (2009). https://doi.org/10.1007/s12010-008-8391-0
Resource availability:	N/A

## Background

Pancreatic cancer remains among the most lethal cancers [1] with a case fatality rate of over 90% [2]. Transgenic mouse models of pancreatic cancer have contributed critical understanding to mechanisms of disease progression and therapeutic resistance characteristic of this aggressive and refractory cancer [3,4]. Next generation sequencing (NGS) technologies are powerful tools to identify dysregulated signaling pathways in cancer, and can identify novel targets and facilitate development of new therapeutic interventions [5]. The ability to extract high quality RNA from mouse pancreatic tumors is therefore necessary to enable the use of advanced RNA sequencing (RNAseq) technology to identify novel pathways and targets that could ultimately improve patient outcomes [6]. Degraded RNA, or poor-quality RNA, can often bias results, and is not suitable for sequencing purposes [7]. In addition to biasing the results, the costs of a failed RNAseq experiment highlight the importance of extracting the highest quality RNA.

RNA extraction is commonly performed in many labs on a daily basis to quantify mRNA levels by using quantitative Polymerase Chain Reaction (qPCR). For qPCR procedures, RNA quality is usually estimated by measuring the A_260_/A_280_ absorbance ratio using a spectrophotometer, where a ratio of ~2.0 represents sufficient RNA purity for qPCR [8]. However, this ratio does not represent the quality of intact RNA, and is not a reliable indicator of RNA integrity for sequencing purposes, as RNA samples with an A_260_/A_280_ ratio of 2.1 can be substantially degraded. Hence, for sequencing purposes, RNA quality and intactness is measured via capillary electrophoresis (CE) and can be quantified using an RNA Integrity Number (RIN) value which ranges from 1 to 10, with 10 representing the highest quality RNA [9], and RNA with RIN values >=8 is suitable for gene expression studies [10]. The major factors that affect the quality of isolated RNA include extraction method, DNA contamination, temperature of the extraction, processing time, and storage. In the current study, we report optimal conditions for extraction of high-quality RNA from mouse pancreatic tumors.

The guanidinium thiocyanate-chloroform extraction method is the most commonly used method for the extraction of RNA from cells and tissues [11]. Although, this method is suitable for extracting high quality RNA from most organs, it is not ideal for pancreatic RNA extraction, as the pancreas is enriched with ribonucleases [12]. Many previously described methods for extraction of high quality RNA from whole murine [13], rat [14,15], and human pancreatic tissue [10] are complex, expensive, and require substantial time and special skills. Most of these reported methods also employ the use of guanidinium thiocyanate, and in the majority of these studies, the RNA quality is assessed using the A_260_/A_280_ ratio [16]. In those prior reports of pancreatic RNA isolation techniques that have employed CE to estimate the quality of RNA, the average RIN value was approximately 9, and the technique involved the perfusion of RNA stabilizing agent into tissue before isolation [6]. More recently, flow cytometry and molecular biology tools were used to isolate cell populations or single cells from pancreas, with resulting RNA RIN values suitable for NGS [17]. Here, we report optimization of a previously reported simple RNA isolation method that reproducibly results in extraction of high-quality RNA (average RIN 9.0) without the use of complex surgical perfusions from mouse pancreatic tissue and tumors.

## Materials & equipment

• 4-month old NSG mice bearing orthotopic pancreatic tumor allografts (IACUC approved)
• Liquid nitrogen
• Guanidinium thiocyanate (TRIzol [Ambion] or equivalent)
• Chloroform (Pharmaco)
• 100% ethanol
• RNaseZap Wipes (Ambion)
• DNase/RNase free pipette tips (VWR)
• Nuclease-free water (Ambion)
• RNeasy mini-plus kit (QIAGEN)
• RNase-Free DNase set (QIAGEN)
• RNA stabilizing agent (Commercial grade; RNAlater [Invitrogen] or equivalent)
• Chemical hood
• Mortar and pestle
• Surgical equipment
• Bioanalyzer 2100 (Agilent)
• Nanodrop 1000 Spectrophotometer (Thermo Scientific)
• Synergy H1 Hybrid Plate reader (BioTek)

## Protocol

**Step 1: Isolation and freezing of Pancreatic tumors**

1.1. The use of animals for the study was approved by the Institutional Animal Care and Use Committee of Virginia Commonwealth University.
1.2. Four-month-old NSG mice were injected with 8 × 10^5^ cells of a mouse pancreatic adenocarcinoma (PDAC) cell line [18] orthotopically into the pancreatic tail vein [19], and the tumor cells were allowed to grow for three weeks before euthanizing the mice.
1.3. Prior to the necropsy and removal of the pancreas, decontaminate the entire workspace and surgical equipment with 70% ethanol and RNase Zap Wipes.
1.4. NSG mice bearing pancreatic tumors were anesthetized using isoflurane and euthanized by cervical dislocation. After euthanizing, the mice were fixed to the dissecting table with the ventral side facing upwards. Sterilize the abdominal area using 70% ethanol, and make an incision in the genital area, cutting open longitudinally to expose the abdomen. Move the bowels towards the left side to get better visualization of the pancreas. As pancreatic tumors are evident, unlike the normal pancreas, make an incision at the common bile duct and isolate the pancreas quickly using scissors and forceps [20]. The whole process need not be carried out under sterile conditions if only for RNA extraction.
1.5 If only a part of the whole pancreatic tumor is needed for RNA isolation, cut the tumor while soaked in RNA stabilizing agent in a petri-dish on ice, then transfer it to a vial/Eppendorf tube and drop in liquid nitrogen to flash freeze the tumor tissue. Transfer the flash frozen tumor vials to −80 °C until extraction.
1.6. Tip: Be as quick as possible and try to maintain cold conditions as much as possible.
1.7. Tip: Mince the tumor or pancreas into small pieces while soaked in RNA stabilizing agent, as this facilitates later steps. Mincing the tumors improves penetration of RNA stabilizing agent and retains integrity of RNA in the tissues.
1.8. Tip on use of RNA stabilizing agent if preferred for long-term storage: Soak the tissue in RNA stabilizing agent before extraction and store as recommended by the manufacturer specifications. We have not compared RNA integrity using RNA stabilizing agent vs. simple snap freezing in liquid nitrogen for the longer-term storage of pancreatic tumors. For next day extraction, snap freezing in liquid nitrogen works effectively and gives the best results.

**Step 2: Extraction of RNA**

**Fig. 1:**
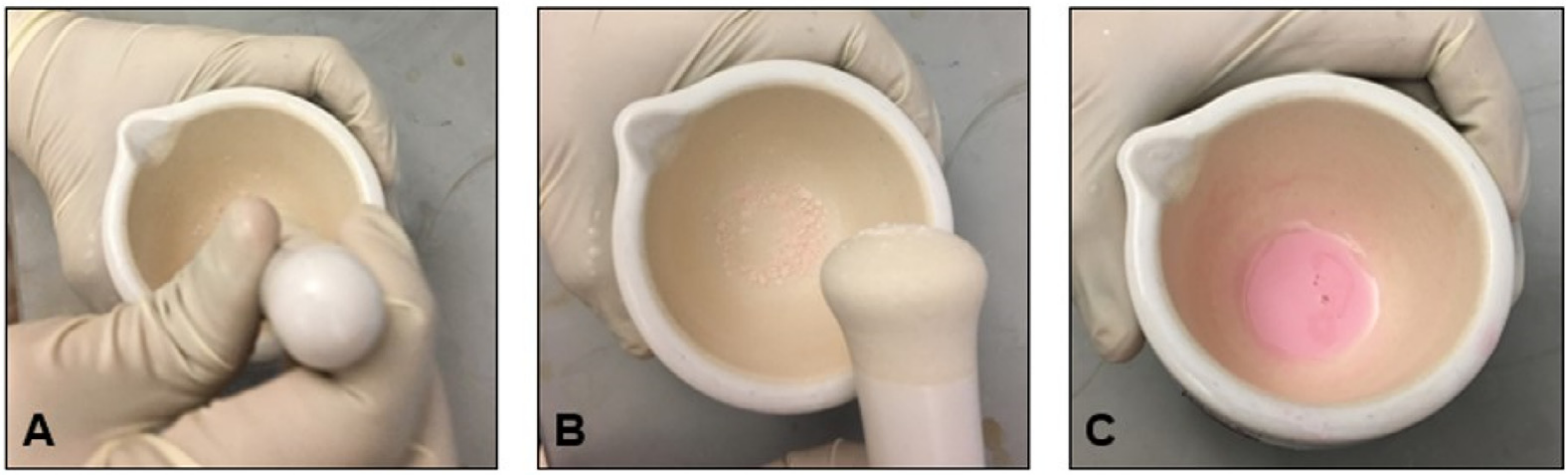
Manual extraction procedure of RNA from mouse pancreatic tumors using liquid nitrogen and TRIzol. (A) Add the pancreatic tumor tissue to the mortar. (B) Powder the tissue by adding liquid nitrogen. (C) Triturate further by adding TRIzol to the powdered tissue.
2.5. Transfer the TRIzol triturate, after it is liquefied, to the DNase/RNase free Eppendorf tube, and incubate for 5 mins at room temperature. It is common to observe solidification of the triturate which liquifies upon on continuing further trituration for a longer time.
2.6. Add 200 µl chloroform per ml of TRIzol lysate and shake vigorously for 15 s. Incubate the tube for 2–3 mins at room temperature before centrifuging in a pre-cooled centrifuge (4 °C) at 11,600 rcf for 15 mins.
2.7. Centrifugation results in separation of the two phases (chloroform aids in separation of the aqueous phase and lower organic phase) in the Eppendorf tube. Now, carefully transfer the upper clear supernatant layer without agitation to a fresh Eppendorf tube and mix with 0.5x volume of 100% ethanol to precipitate nucleic acids (DNA, RNA).
2.8. Tip: Try not to disrupt the bottom solvent layer, as this will result in blocking the RNA spin column and decreases the efficiency of the method.

2.1. Before performing extraction, clean the mortar and pestle using RNaseZap Wipes.
2.2. Place pestle in mortar and add liquid nitrogen to cool both, and place TRIzol solution on ice or use it immediately after removing from refrigerator.
2.3. Tare the weighing machine using an empty weighing boat and transfer the pancreas/tumor tissue from the flash frozen vial into a weighing boat and record the weight of the tissue. Now quickly transfer the tissue to the mortar, and powder the tissue while maintaining liquid nitrogen in the mortar to keep everything cold and facilitate crushing of the tissue into a frozen powder. Do not allow the tissue to thaw.
2.4. After the evaporation of the liquid nitrogen, add TRIzol in 500µl aliquots to the powdered tissue, with 500 µl for every 50 mg of pancreas/tumor tissue, and continue trituration as shown in Fig. 1**.** We recommend up to 1 ml of TRIzol per 100 mg of tumor tissue for best yields. Carry out trituration in a chemical hood to avoid hazardous health effects of the TRIzol.

**Step 3: Purification and Isolation of RNA**

3.1. After mixing with ethanol, transfer the mixture to the QIAGEN RNeasy mini spin column and centrifuge at 9600 rcf for 30 s.
3.2. Wash the column using 350 µl RW1 wash buffer from the RNeasy mini extraction kit.
3.3. Add 40 µl of freshly prepared DNase I solution (Thermo Fisher) and incubate the column for 15 min at RT, after which, wash the column with RW1 wash buffer for the second time and centrifuge at 9600 rcf for 30 s. This eliminates DNA contamination of the extracted RNA.
3.4. Discard the flow through, and wash the column with 500 µl RPE buffer supplied in the kit two times at 9600 rcf for 30 s.
3.5. Finally, spin the column at 16,000 rcf for 2 min to remove any remaining RPE buffer from column. Make sure you remove the RPE buffer completely for better results.
3.6. Add 30–40 µl of RNase free water to the column and incubate the column for 1 min at room temperature.
3.7. Centrifuge at 9600 rcf for 30 s to collect the pure RNA.
3.8. Tip: Avoid including tissue pieces in the solution that might result in blocking of the column and reduce column efficiency.
3.9. Tip: As the binding affinity of the RNase easy spin columns is limited, we recommend dividing the total extract into equal volumes and using multiple columns to avoid clogging of the columns and combine the eluates after isolation to restore sufficient volume.
3.10. Tip: Avoid repeated elution, as most of the RNA obtained by repeated elution is not high enough quality for sequencing purposes.

**Step 4: Quantification of RNA**

4.1. The concentration of RNA is measured using a Nanodrop (Thermo Scientific, USA) by loading 2 µl of the sample.
4.2. The concentration and the absorbance ratio A_260_/A_280_ value obtained in the Nanodrop gives an estimate of concentration and any protein contamination.
4.3. However, 260/280 value is not a determinant of intact RNA, so make sure you have sufficient RNA for the quality check and sequencing steps.

**Step 5: Quality Check**

**Fig. 2:**
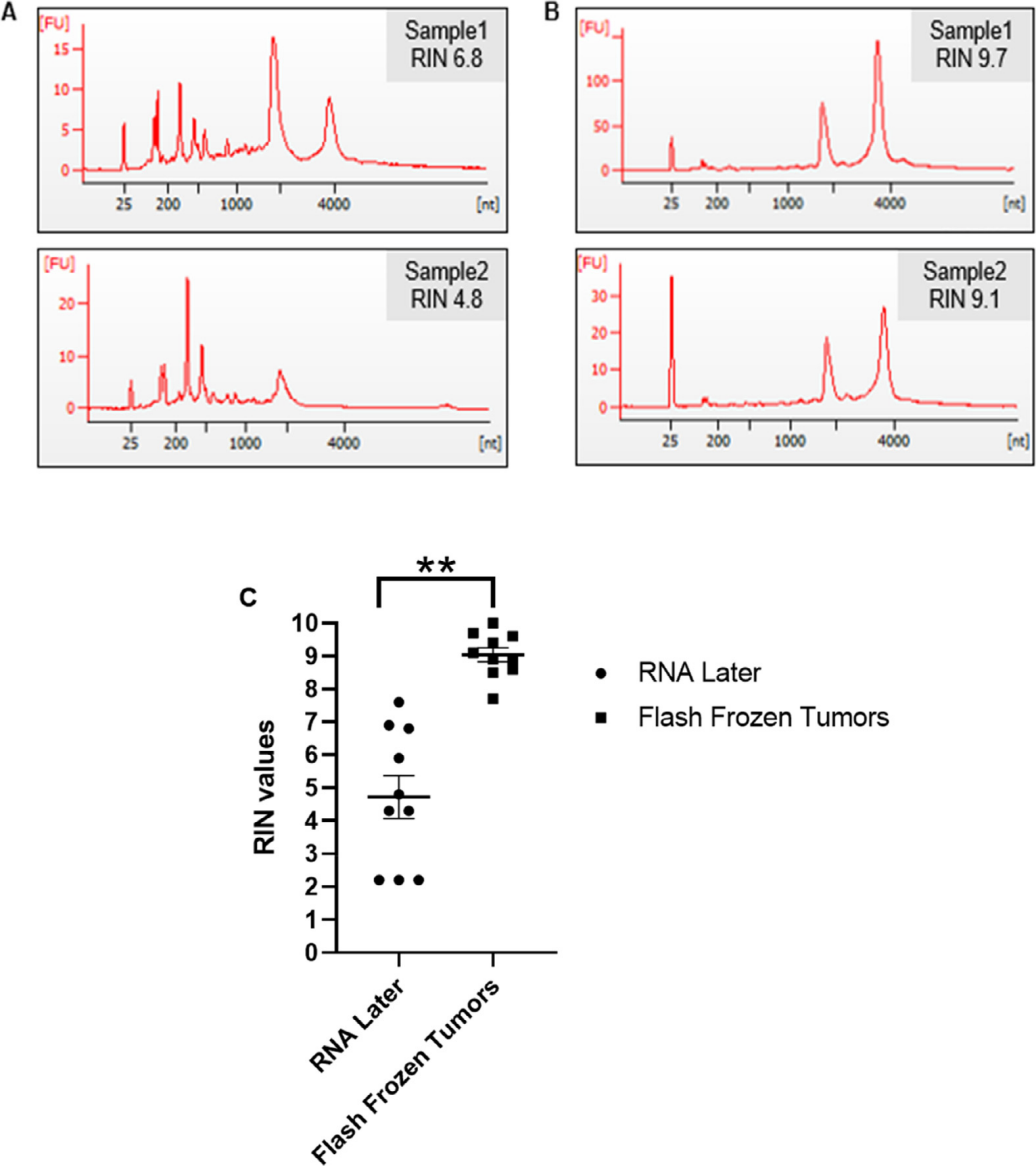
Extraction after flash freezing maintains RNA integrity. The integrity of representative RNA samples (RIN values) isolated from pancreatic tumors: A) Soaked in RNA stabilizing agent and extracted with TRIzol on ice; or B) Flash frozen and powdered using liquid nitrogen and then extracted with TRIzol. C) RIN values for RNA isolated from pancreatic tumors (*n* = 10/group) using techniques in (A; “RNA Later”) and (B; “Flash Frozen Tumors”). Mean RIN values were 4.7 (A) and 9.0 (B) **p< 0.01 (paired *t*-test). Error bars represent SEM.
5.2. The extracted RNA is stored at −80 °C till use for sequencing.
5.3. Tip: Run the RNA samples on an agarose gel as described [21] and examine for 18 s and 28 s rRNA bands before sending samples for Bioanalyzer quality check to quickly determine if it is worth analyzing the samples.
5.4. Tip: To avoid multiple freeze thaws, divide the RNA isolate into multiple aliquots and store at −80 °C. Use each aliquot once.
5.5. Tip: Use dry ice to transfer the samples for quality check or sequencing to protect RNA integrity.

5.1. Check the integrity of the RNA using a Bioanalyzer 2100 instrument (Agilent), following the standard protocol for running total eukaryotic RNA on a pico chip, to determine the RIN of the sample (Fig. 2).

**Step 6: Storage**

6.1. The extracted RNA is stored at −80 °C till use.
6.2 Tip: It is advisable to use the RNA as quickly as possible for sequencing purposes to avoid any possible degradation.

## Method validation

Although numerous methods have been published which describe the extraction of high-quality RNA from pancreas or pancreatic tumors, our method is simple, reliable and reproducible, resulting in extraction of high-quality RNA (mean RIN = 9.0) from mouse pancreatic tumors that is especially well-suited for NGS analyses. Incorporation of these simple tips will help greatly in improving the quality of RNA and make pancreatic-derived RNA more consistently usable for sequencing purposes, saving money and avoiding bias in the sequencing results. With further adaptation, we envision this technique being applicable to the isolation of high-quality RNA from human pancreatic normal and tumor specimens as well.

## Declaration of Competing Interests

The authors declare that they have no known competing financial interests or personal relationships that could have appeared to influence the work reported in this paper.
